# Analyzing complications and implementing solutions in a pediatric inguinal hernia cooperation program in Equatorial Guinea: a prospective cohort study

**DOI:** 10.1186/s43159-022-00237-5

**Published:** 2023-01-10

**Authors:** Jaime Rodríguez de Alarcón García, Amalia Úbeda Pascual, María Fanjul Gómez, Pablo Morató Robert, Rocío Espinosa Góngora, Ernesto Martínez García, Carlos Román Guerrero, Santiago Jaime Abaga Abaga, Carmen Soto Beauregard

**Affiliations:** 1 Department of Pediatric Surgery, Hospital Clínico San Carlos, Madrid, Spain; 2 Medical Faculty, Universidad Francisco de Vitoria, Madrid, Spain; 3 Department of Pediatric Surgery, Hospital Universitario Gregorio Marañón, Madrid, Spain; 4 Department of Pediatric Surgery, Hospital Infantil Universitario Niño Jesús, Madrid, Spain; 5 Department of Anesthesiology, Hospital Infantil Universitario Niño Jesús, Madrid, Spain; 6 Department of Anesthesiology, Hospital Universitario de la Princesa, Madrid, Spain; 7 Medical Center, SOS Children’s Villages, Bata, Equatorial Guinea

**Keywords:** Short-term medical mission, Pediatric inguinal hernia, Pediatric surgery cooperation program, Complication rates, Surgical site infection

## Abstract

**Background:**

Few studies have evaluated the efficacy of short-term medical missions. This study was aimed to evaluate complication rates and determine the effects of protocol changes in a pediatric inguinal hernia campaign in Equatorial Guinea and analyze post-operative follow-up capacity.

**Methods:**

In this prospective observational cohort study, we evaluated two patient cohorts (group A, 2017–2018; group B, 2019) treated during campaigns in Equatorial Guinea for congenital inguinal pathology (hernia, hydrocele, and cryptorchidism). Patients aged < 18 years treated in referral campaigns were included. Complications occurring up to 6 months post-operatively were evaluated. Two stages were defined: Stage 1, wherein, complication rate in group A was compared to that in a control group from a tertiary hospital in Spain (with a case–control ratio of 1:2, paired according to age, sex and diagnosis); stage 2, wherein, complication rates between groups A and B were compared. Group B received a single dose of prophylactic amoxicillin-clavulanic acid. Follow-up capacity was assessed through follow-up appointments.

**Results:**

In stage 1, complication and surgical site infection (SSI) rates were 21.3% and 7.4% in group A (*n* = 94), and 5.8% (*p* < 0.001) and 0.5% (*p* = 0.012) in the control group, respectively. Group A had 20.2% loss-to-follow-up. In group B (*n* = 62), 6-month postoperative follow-up could not be assessed owing to restrictions due to the COVID-19 pandemic, so only early complications were considered in stage 2, were complication and surgical site infection rates were 18.1% and 7.4% in group A and 11.3% (*p* = 0.350) and 1.6% (*p* = 0.150) in group B.

**Conclusion:**

Our results showed higher than expected complication rates. Pre-operative prophylactic antibiotic could not show to reduce SSI. Further studies are needed to reduce complication rates in these campaigns. Patient loss-to-follow-up ratio warrants considering new strategies.

## Background

In short-term medical missions (STMMs), physicians, otherwise fully employed in their countries, spend short periods in lower- and middle-income countries (LMICs) providing unpaid service [1]. However, only a few studies investigating STMM efficacy have been published, and they lack consistent terminology. Martiniuk et al. [2] reviewed studies concerning STMMs published between 1985 and 2009 in LMICs. Of 2512 studies, 230 were analyzed, with most being descriptive studies (74%) that seldom addressed issues such as ethical conflicts or the evaluation of clinical outcomes.

Without questioning the humanitarian value of these campaigns, it is important to determine the clinical efficacy of STMMs and evaluate the quality of care provided. Such analyses should also serve to identify any programmatic weaknesses and refine improvement strategies.

Our group has been conducting surgical cooperation campaigns in Equatorial Guinea for > 15 years in collaboration with the non-governmental organization, SOS Children’s Villages. These expeditions focus on resolving frequent pediatric pathologies that generate morbidity or disability, are relatively simpler to treat, and have few complications. We have mainly treated inguinal hernia and its associated conditions (hydrocele and cryptorchidism). Inguinal hernia is a highly prevalent condition, especially in Africa [3], and several studies have shown that inguinal hernia surgery programs are cost-effective [4]. Saxton et al. [5] showed in a systematic review of children surgical care in LMICs that inguinal hernia repair has the lowest cost effectiveness ratio, considering that inguinal hernia repair should be considered an essential children’s surgical procedure based on its great economic value. Other studies shown also that can be delivered at the appropriate quality standards and have a relevant effect on the quality of life [6].

The main goal of this study was to evaluate complication rates in hernia and related conditions surgery in a pediatric age group and establish and analyze strategies to reduce them. The secondary goal was to assess the follow-up capacity of the patients enrolled in the cooperation program**.**

## Patients and methods

We conducted an analytical observational prospective cohort study with two patient cohorts (group A [*n* = 94 patients; years 2017–2018] and group B [*n* = 62 patients; year 2019]) treated during cooperation campaigns undertaken at the SOS Children’s Village facilities in Bata, Equatorial Guinea.

All patients aged < 18 years who had been treated during 2017, 2018, and 2019 in the referred campaigns for uncomplicated congenital inguinal pathology (hernia, hydrocele, and cryptorchidism) were included. Exclusion criteria comprised children with generalized debilitating disease, infective focus, or fever.

**Fig. 1:**
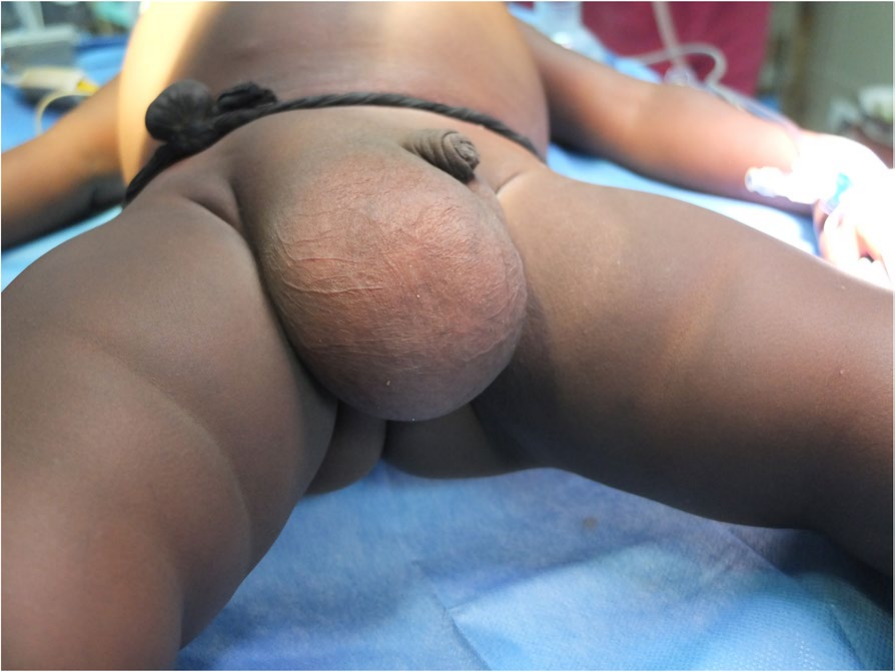
Giant hernia

**Fig. 2:**
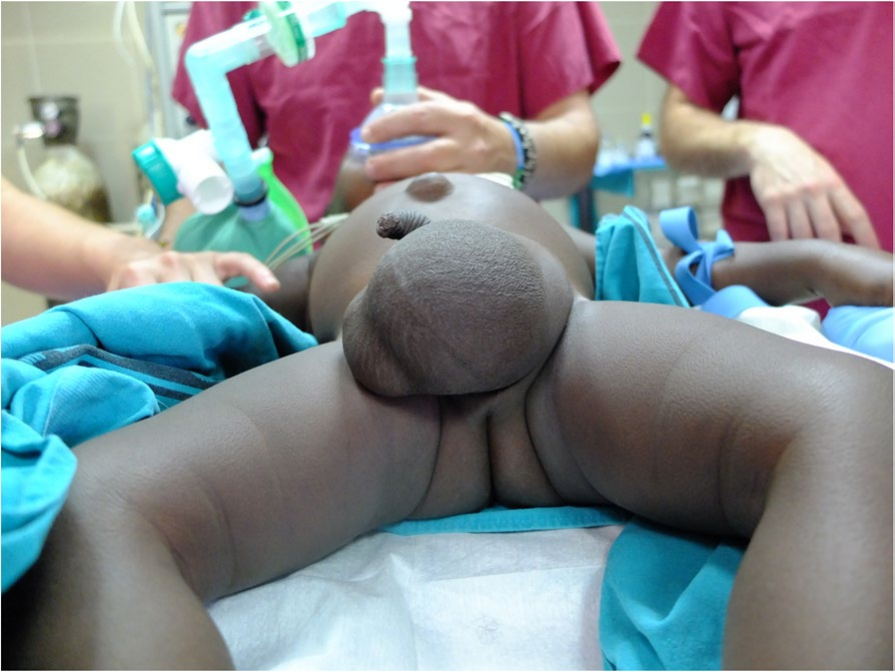
Giant hernia

Demographic (age, sex) and anthropometric (weight, height) parameters were recorded. According to age, patients were divided into four groups: ≤ 12 months, > 12 months to ≤ 5 years, > 5 years to ≤ 10 years, and > 10 years to ≤ 18 years. To assess nutritional status, weight size ratio percentile (pWS) was obtained in patients younger than 5 years and divided into three categories: obesity if pWS is ≥ 90, normal for pWS between 90 to ≥ 10, and malnourished for pWS of < 10. In patients older than 5 years, body mass index percentile (pBMI) was calculated, and patients were categorized into three groups: Obesity (pBMI of ≥ 97), normal (pBMI of 97 to ≥ 10), and malnourished (pBMI of < 10). This categorization was made based on World Health Organization 2006/2007 tables [7] and according to available guidelines [8]. The pre-operative examination included serology for malaria, human immunodeficiency virus (HIV), hepatitis B virus (HBV), and hepatitis C virus (HCV), and determination of hemoglobin levels (Hb). Additionally, any relevant previous medical history and adherence to the local vaccination schedule were noted. If an inguinal hernia or hydrocele was significantly larger than that observed in usual practice in Spain, as assessed by two different surgeons, this was also recorded (Figs. 1 and 2).

Complications were assessed intra-operatively, prior to discharge (24-h post-operatively), and at 7-day and 6-month follow-ups. Parents were instructed to make an unscheduled visit if there was any sign of complications, and unscheduled visits were also noted. If a patient did not attend a follow-up appointment, contact was made through telephone. Complications were categorized as *intraoperative* if happened during surgery, *early* if occurred during the first week and *late* if happened after the 7th day. Loss-to-follow-up (early or late) was also noted, and follow-up capacity was evaluated. Early post-operative complications were defined as surgical site infection (SSI), symptomatic hematoma, symptomatic hydrocele, post-operative disabling pain, or significant post-operative nausea/vomiting, during the first week after surgery. Possible late complications were recurrence, hydrocele, hypertrophic/keloid scarring, or testicular atrophy.

All patients with inguinal hernias underwent a standard open approach, involving high ligation of the hernia sac and Ferguson repair of the inguinal canal, if needed, using absorbable sutures [9]. For orchidopexy, the Shoemaker technique was performed [10]. General anesthesia with locoregional or caudal epidural block depending on age was performed in every patient. The operating facilities were set up by the members of the expedition and all effort was made to comply with the best standards of practice.

A two-stage study was conducted. In stage 1, we analyzed group A’s complication rate and was compared with a cohort of historical controls from Hospital Clínico San Carlos in Madrid, Spain (control group). Control group data were obtained through a review of relevant medical records. A case–control ratio of 1:2 was considered and matched according to age group: ≤ 12 months, > 12 months to ≤ 5 years, > 5 years to ≤ 10 years, > 10 years to ≤ 18 years; sex; and pathology. For bilateral involvement, a control with similar characteristics was chosen. Controls were selected through non-randomized sampling and choosing the first patient who had undergone surgery in the last 4 years who met the matching criteria, who had at least 6 months of follow-up, and who had not been previously selected. Hemoglobin levels, nutritional status and infectious diseases were not available for control group, since it is not considered at this center protocol for ambulatory procedures in otherwise healthy patients. Regarding surgical technique the same procedures were used for each diagnosis in groups A, B and Control group.

In stage 2, we compared group A and B complication rates, in latter group the peri-operative protocol was modified to include the administration of a prophylactic dose of pre-operative antibiotic (intravenous amoxicillin-clavulanic acid, 30 mg/kg), which was not administered in group A and the control group, according to the available evidence and guidelines [11].

Data analysis was performed using SPSS version 11.1 for Windows software (SPSS Inc., Chicago, IL, USA). A two-sample chi-square test was used to compare the groups. Statistical significance was set at a *P* value of ≤ 0.05.

## Results

Group A comprised 94 patients (males, 84%). The largest age group (1–5 years) accounted for 41.49% of the total patients, and 52.13% had unilateral hernia. Fourteen patients had hernias or hydroceles considered larger than usual. The incidence of malnourishment and obesity was 7.45% and 36.17%, respectively. Regarding infectious diseases, malaria was diagnosed and treated preoperatively in 25.53% of patients in group A. Anemia, defined as hemoglobin levels < 11 g/dL in 6 to 59 month age group; < 11.5 g/dL in 5 to 11 years group; < 12 g/dL in 12- to 14-year group; < 12 g/dL in non-pregnant women over 15 years old and < 13 g/dL in males older than 15 years old [12], was found in 53.19% of patients in group A (Table 1). About 60.6% had complete accomplishment of vaccine calendar.

**Table 1:** Comparison of demographics between groups A and B

Characteristics	Group A (*n* = 94)	Group B (*n* = 62)	*P* value A vs B	Control group (*n* = 188)
Males	79 (84.04%)	51 (82.26%)	0.770	158 (88.04%)
Malnourished	7 (7.45%)	6 (9.68%)	0.79	N/A
Overweight	34 (36.17%)	12 (19.35%)		N/A
Anemia	50 (53.19%)	41 (66.13%)	**0.032**	N/A
Malaria	24 (25.53%)	14 (22.58%)	0.674	N/A
HIV	1 (1.06%)	0 0%	0.523	N/A
HBV	4 (4.26%)	2 (3.23%)	1.000	N/A
HCV	2 (2.13%)	0 0%	0.518	N/A
*Age group*				
< 12 months	7 (7.45%)	6 (9.68%)	0.275	14 (7.45%)
1–5 years	39 (41.49%)	27 (43.55%)		78 (41.49%)
> 5 to 10 years	32 (34.04%)	13 (20.97%)		64 (34.04%)
> 10 years	16 (17.02%)	16 (25.81%)		32 (17.02%)
*Diagnoses*				
*Inguinal hernia*				
Unilateral	49 (52.13%)	38 (61.29%)	0.336	98 (52.13%)
bilateral	7 (7.45%)	5 (8.06%)	0.887	14 (7.45%)
*Hydrocele*				
Unilateral	17 (18.09%)	10 (16.13%)	0.752	34 (18.09%)
Bilateral	3 (3.19%)	0 0%	0.277	6 (3.19%)
Giant hernia or hydrocele	14 (14.89%)	4 (6.45%)	0.106	0
*Cryptorchidism*				
Unilateral	18 (19.15%)	0 (0%)	0.133	36 (19.15%)
Bilateral	6 (6.38%)	3 (4.84%)	0.061	12 (6.38%)

*HIV* Human immunodeficiency virus, *HBV* Hepatitis B virus, *HCV* Hepatitis C virus

*N/A* No data available

Stage 1 compared the complication rates in group A with those in a control group from a tertiary center in Spain. Group A had an overall complication rate of 21.28%, compared with 5.85% of the control group (*p* < 0.001). The SSI rates in group A and the control group were 7.45% and 0.53%, respectively (*p* = 0.012). Seven patients in group A developed SSIs during the first 7 days post-operatively, only one of them had undergone bilateral surgery (Table 2). In the < 12-month age group, two of seven patients presented with SSIs. Of those assessed as having large hernias or hydroceles (*n* = 14), five patients (36%) had complications (SSI, *n* = 2; hematoma, *n* = 2; deferential injury, *n* = 1).

**Table 2:** Complication incidence distributed between study groups

Complications	Control group (*n* = 188)	Group A (*n* = 94)	Group B^a^ (*n* = 62)
Early			
Intraoperative			
Iatrogenic vasectomy	0 (0%)	2 (2.13%)	0 (0%)
During first week			
SSI	1 (0.53%)	7 (7.45%)	1 (1.61%)
Hematoma	2 (1.06%)	5 (5.32%)	4 (6.45%)
Wound dehiscence	1 (0.53%)	2 (2.13%)	0 (0%)
Other (pain, inflammation)	2 (1.06%)	1 (1.06%)	2 (3.23%)
Late			
Relapse	3 (1.6%)	2 (2.13%)	N/D
Hypertrophic scar	2 (1.065)	1 (1.06%)	N/D
Total	11 (5.85%)	20 (21.28%)	7 (11.29%)

*SSI* Surgical site infection

^a^Antibiotic prophylaxis

Regarding follow-up, eight patients (8.51%) in group A did not attend the 7-day post-operative follow-up appointment; however, these patients were contacted via the telephone, and they reported no major complications. Twenty-one patients (22.34%) did not attend the 6-month follow-up appointment and could not be contacted.

Group B comprised 62 patients (82.26% males) treated during the campaign of 2019. A preoperative antibiotic prophylaxis with amoxicillin-clavulanic acid was administered to every patient during anesthetic induction, as the selected strategy to reduce SSI rate. As observed in Group A, the largest age group (1–5 years) which included 43.55% of patients, had unilateral hernia as the most common diagnosis, including 61.29% of patients. Four patients had large hernias or hydroceles (Table 1). Approximately 9.68% of patients were considered malnourished and 19.35% were overweight. Malaria was also the most common infectious disease in group B, with 22.58% of patients diagnosed and treated preoperatively, 66.13% had anemia, and 30.65% accomplished the vaccine calendar, while the rest of them had an unknown or uncomplete status.

In group B, all patients attended the first follow-up appointment. The second follow-up appointment was canceled, given the impossibility of undertaking the 2020 campaign owing to restrictions due to the COVID-19 pandemic. Table 1 presents the comparison between group A and B demographics, pathology, and diagnosis. Comparing both groups, only anemia shown a statistically significant rate.

At stage 2, complication rates between groups A and B were compared. As group B couldn’t be assessed for late complications in the second follow-up appointment, only those during the first week were included.

**Fig. 3:**
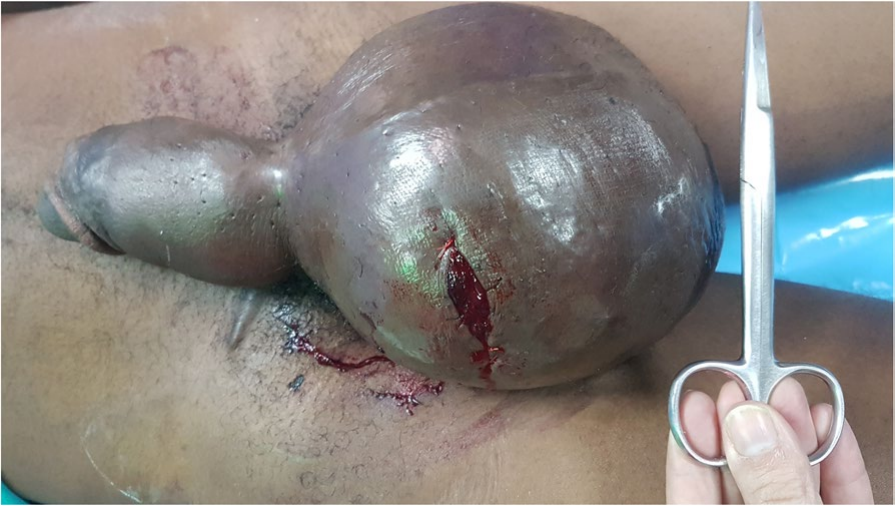
Hematoma in a 16-year-old boy after hidrocelectomy, which required surgical drainage

Early complication rates were 18.08% in group A and 11.29% in group B (*p* = 0.350). SSI rates were 7.45% in group A and 1.61% in group B. The addition of antibiotic prophylaxis in Group B did not achieved a statistical significance between SSI rates of groups (*p* = 0.150), despite a reduction in the absolute frequency of complications and SSI rates were observed in this group B. Four patients presented with symptomatic hematoma in group B, one of whom required surgical treatment (Fig. 3).

The overall incidence of complications in each group is summarized in Table 2.

## Discussion

Inguinal hernia, hydrocele, and cryptorchidism are similar entities with patency of the processus vaginalis as a common feature [13]. Furthermore, 26% of cryptorchidic testicles are associated with inguinal hernia [14] and 25% with hydrocele [15]. The surgical approaches are similar, involving dissection and proximal ligation of the processus vaginalis after separating it from the spermatic cord, or the round ligament in the case of a female patient with a Nuck cyst [16].

Together, these common surgical conditions in children are an important cause of morbidity and disability [5]. The overall incidence of inguinal hernia ranges from 0.8 to 5.0%, increasing to 30% in preterm infants [17]. Several studies have suggested that this incidence rate may be higher in sub-Saharan African populations [18, 19]; however, no robust evidence has been published to support this suggestion [3].

Inguinal hernia repair is a frequent target of STMMs, as it is a common condition affecting quality of life. It is relatively simple to correct, involving a cost-effective procedure, even in low-resource scenarios [5], with few reported complications and can be undertaken to accomplish appropriate high-quality standards [19].

This study showed a higher complication rate than expected, especially in group A, with an SSI rate of 7.45%. Excluding SSI and late complications that could not be assessed in group B owing to restrictions due to the COVID-19 pandemic, similar early complication rates were observed in both groups (group A, 10.63%; group B, 9.67%), which were higher than found in our control group (2.65%), meaning that the major impact in those differences may be due precisely to SSI rate. In group A, two of seven patients from the < 12 months age group (29%) developed SSI. This may be related to the use of diapers and worse surgical wound hygienic care in these patients. The same age group with a similar number of patients presented no SSI in group B, where antibiotic prophylaxis was administered as the finally implemented strategy in order to reduce the most frequently reported complication in group A. Five of 14 patients (36%) with “big” hernias or hydroceles had complications in group A, while none of the four patients considered in group B had any complication. It remains unclear whether this could be related to antibiotic prophylaxis or due to the small sample size; further studies with larger groups should specially consider younger children with larger hernias or hydroceles. Those “big” hernias could have higher complication rates due necessity of wider dissection and bigger skin incisions. Given the absence of giant hernias in control group, the role of hernia size in complication rates remains unclear.

Vas deferens injury is uncommon in our practice and there were no cases observed in the control group. Two patients in group A who had iatrogenic vasectomy that was noticed and repaired during surgery, and it was the only intraoperative complication recorded. This may be attributed to the surgeons’ fatigue owing to long working hours for many days or particularly challenging cases.

The sample size in this study is insufficient to determine if any other demographic, biometric, or analytical parameters have a direct effect on complication or SSI rates. Bucher et al. [20] suggested that developmental, socioeconomic, and genetic parameters could be involved in higher SSI rates in some patients.

Published complication rates for pediatric inguinal hernia differ substantially, probably due to varying conditions in each study and different definitions of complications (Table 3) [21–27]. General complication rates range from 1.4 to 17.0% in LMICs, but studies related to complicated hernias in preterm or newborn patients have reported even higher complications and SSI rates [22, 23]. Also, group A showed a higher SSI rate than other studies form LMICs.

**Table 3:** Complication rates reported in different studies and rates found in this study

	Omar et al	Nagraj et al. ^a^	Bamigbola et al.^b^	Erdogan et al	Askapour et al	Javaid Et al	Chu et al. ^c^	Control group	Group A	Group B^d^
Year	2004	2006	2012	2013	2013	2018	2019	2021	2017–2018	2019
Country	Libya	UK	Nigeria	Turkey	Iran	Pakistan	China	Spain	Eq. Guinea	Eq. Guinea
Number of patients	827	125	41	3776	269	241	3006	188	94	62
Complication rate	6.6%	18.4%	24.4%	12%	5.2%	17.01%	1.4%	5.8%	21.3%	11.2%
SSI	1.9%	2.3%	14.4%	0.6%	0.4%	0%	0.3%	0.5%	7.5%	1.6%

^a^Weight < 5 kg

^b^Complicated hernias

^c^Laparoscopic or open surgery with patch

^d^Antibiotic prophylaxis

Only few reports compare hernia repair results from cooperation campaign in LMICs with data obtained from higher income countries. Gil et al. [19] compared different effectiveness and quality indicators in campaigns in Cameroon and Mali with a cohort from a tertiary center in Spain in adult populations. Despite the heterogeneity of groups and lack of health infrastructure in the African setting, they described similar complication rates and clinical outcomes. Some opportunities for improvement were considered, like increasing the follow-up at discharge by local health agents. To the best of our knowledge, no previous results on STMM have been published regarding pediatric inguinal hernia; therefore, a comparison using previous studies could not be undertaken, given the very particular conditions of our group. We considered the goal of complication rates in our mission to be the same that those in our regular practice in the most similar patient cohort, as done in this study.

Given the high complication and SSI rates found in this study, further efforts to reduce these, particularly, the SSI rate, were proposed. At clean pediatric surgical procedures, the risk of SSI is extremely low, the unnecessary use of antibiotics in children could cause deleterious adverse events and promote antimicrobial resistance, so according to current evidence and guidelines, prophylactic antibiotics are not recommended for pediatric herniotomy or orchiopexy [28, 29], but after the preliminar analysis of group A antibiotic prophylaxis was considered to be included in the protocol. Zamkowski et al. [30] recommended that antibiotic prophylaxis should be considered even in low-risk patients if SSI rates above 4% are found. Previously published studies have not provided high-quality evidence concerning antibiotic prophylaxis use. Osuigwe et al. [31] published a randomized double-blinded study to evaluate the need for prophylactic antibiotics in pediatric day-case surgery in Nigeria; the study showed 4.3% and 5% SSI rates with and without antibiotics, respectively. In a randomized prospective study in India, the SSI rate was 3.73% and 2.22% in the case and control groups, respectively (*P* = 0.702) [32]; however, another study suggested that, under certain conditions, antibiotic prophylaxis in combination with occlusive dressings would likely prevent SSI [33]. In a systematic review, Murni et al. [34] found that the most effective measure to reduce nosocomial infection was the implementation of hand hygiene campaigns, which we will also implement in further campaigns. Cooper et al. [35] concluded that interventions to prevent SSI should be adapted to the local context considering particular conditions, such as local antimicrobial resistance or education of patients regarding antibiotic use.

In this study, the SSI rate was reduced from 7.5 to 1.6% with antibiotic prophylaxis use. While not statistically significant, maybe due to insufficient study sample, so we intend to include larger patient groups in further studies to determine the effect of this intervention more clearly. Other measures, such as hand hygiene campaigns, the use of triclosan coated sutures [36], or changing the surgical dressing protocol, also must be evaluated.

Regarding follow-up, 20% of patients in group A did not attend the 6-month appointment, and we were unable to contact these patients via the telephone. While the complication rate at 6 months was low (recurrent hydrocele, *n* = 2; hypertrophic scarring, *n* = 1), the relatively high loss-to-follow-up rate indicates that we should consider changing the follow-up protocol before including more complex pathologies in our program for which closer or longer follow-up times would be required. Shorter time between appointments may lead to better outcomes.

This study is limited by the number of patients included in each group. COVID-19 had a relevant impact also in cooperation abroad programs, and our campaigns are temporary stopped, so we could not assess long-term follow-up or include more patients in group B as was initially considered. Larger groups could help to determine the role of antibiotic prophylaxis to prevent SSI, as the contribution of demographic or anatomical factors on complication rates. Also, working in a foreign environment with limited resources along with language and social barriers makes it difficult to collect and analyze other factors that may affect complication rates, such as personal hygiene, socioeconomic status, or surgical wound care at home. Further studies should also consider even closer collaboration with local agents to enable identification of patients with higher complication rates risk and to focus efforts on their prevention.

## Conclusion

While STMMs are undertaken in more challenging conditions, clinical data collection and analysis are feasible and desirable. The high complication rate found in this study has led us to review and adapt specific protocols. Antibiotic prophylaxis did not significantly affect SSI rates in our study; however, further studies with larger groups are needed to confirm this result. The loss-to-follow-up rate in this campaign was high. Therefore, the implementation of patient loyalty programs is needed to assess long-term complications.

## Data Availability

The authors confirm that the data supporting the findings of this study are available from the corresponding author upon request.

## Abbreviations

SSI: Surgical site infection
STMMs: Short-term medical missions
LMICs: Lower- and middle-income countries
pWS: Weight size ratio percentile
pBMI: Body mass index percentile
HIV: Human immunodeficiency virus
HBV: Hepatitis B virus
HCV: Hepatitis C virus
Hb: Hemoglobin
